# Marine Bioactive Peptides: Anti-Photoaging Mechanisms and Potential Skin Protective Effects

**DOI:** 10.3390/cimb46020063

**Published:** 2024-01-23

**Authors:** Xiaoliang Zhang, Hong Zhuang, Sijia Wu, Chen Mao, Yaxi Dai, Haiyang Yan

**Affiliations:** College of Food Science and Engineering, Jilin University, Changchun 130062, China; zhangbg_research@163.com (X.Z.); wusijia2017@sina.com (S.W.); maochen_workmail@163.com (C.M.); daiyx21@mails.jlu.edu.cn (Y.D.); yanhy@jlu.edu.cn (H.Y.)

**Keywords:** skin photoaging, anti-photoaging, peptides, marine bioactive peptides

## Abstract

Skin photoaging, resulting from prolonged exposure to ultraviolet radiation, is a form of exogenous aging that not only impacts the aesthetic aspect of the skin but also exhibits a strong correlation with the onset of skin cancer. Nonetheless, the safety profile of non-natural anti-photoaging medications and the underlying physiological alterations during the process of photoaging remain inadequately elucidated. Consequently, there exists a pressing necessity to devise more secure interventions involving anti-photoaging drugs. Multiple studies have demonstrated the noteworthy significance of marine biomolecules in addressing safety concerns related to anti-photoaging and safeguarding the skin. Notably, bioactive peptides have gained considerable attention in anti-photoaging research due to their capacity to mitigate the physiological alterations associated with photoaging, including oxidative stress; inflammatory response; the abnormal expression of matrix metalloproteinase, hyaluronidase, and elastase; and excessive melanin synthesis. This review provides a systematic description of the research progress on the anti-photoaging and skin protection mechanism of marine bioactive peptides. The focus is on the utilization of marine bioactive peptides as anti-photoaging agents, aiming to offer theoretical references for the development of novel anti-photoaging drugs and methodologies. Additionally, the future prospects of anti-aging drugs are discussed, providing an initial reference for further research in this field.

## 1. Introduction

The examination of marine bioactive peptides has garnered significant interest over the past two decades owing to their extensive occurrence in various marine sources and their notable biological effectiveness [1]. These peptides are primarily derived from mollusks, crustaceans, fish, algae, and certain marine by-products (such as shellfish, fish skin, offal, and muscle) [2,3]. The investigation of marine bioactive peptides has received substantial attention in recent years, primarily due to their widespread presence in diverse marine sources and their potent biological activity [4]. Currently, Peptides derived from marine sources are employed for their advantageous biological properties, including but not limited to anti-aging [5], anti-oxidant [6], anti-inflammatory [7], anti-microbial [8], anti-hypertensive [9], and anti-tumor activities [10]. Moreover, peptide compounds are extensively utilized in the advancement of various novel food, cosmetic, and pharmaceutical products owing to their minimal toxicity, functional versatility, specificity, and wide-ranging efficacy [3,11]. Notably, marine bioactive peptides have been documented to mitigate the likelihood of photoaging induced by UV radiation, thereby exerting a regulatory influence on skin aging [3,12]. Hence, marine bioactive peptides possess promising potential in the realm of skin protection.

Skin aging is a multifaceted phenomenon encompassing both endogenous and exogenous processes [13]. Among the external factors, ultraviolet (UV) radiation stands out as the primary contributor to skin photoaging [14]. Currently, the predominant approaches to counteract photoaging involve pharmaceutical interventions, physical and chemical therapies, and surgical interventions [15,16]. One such pharmaceutical intervention is retinoic acid, which has received approval from the U.S. Food and Drug Administration (FDA) for the treatment of skin photoaging [17]. However, its application is constrained by the occurrence of adverse reactions, such as burning, flaking, and dermatitis. Hence, the exploration of innovative pharmaceutical candidates pertaining to anti-photoaging mechanisms has emerged as a prominent subject of scientific inquiry. Marine bioactive peptides, originating from the metabolites of marine organisms, constitute a significant component of the human dietary intake [18,19]. Nevertheless, research investigating the anti-photoaging properties of peptides remains limited, thereby impeding their potential application within industries focused on enhancing skin quality. Nonetheless, numerous conjectures and perspectives exist regarding the underlying mechanisms and pathways associated with these peptides.

**Figure 1:**
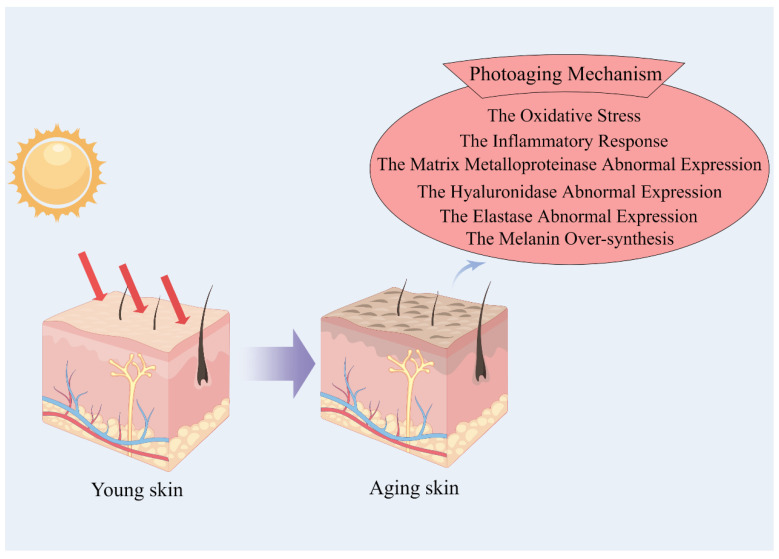
The factors in photoaging.

Research has demonstrated that marine bioactive peptides play a significant role in numerous anti-photoaging hypotheses [20], encompassing the oxidative stress theory [21,22,23,24,25,26,27,28,29,30,31,32,33,34,35,36,37], the inflammatory response theory [38,39,40], the matrix metalloproteinase abnormal expression theory [41,42,43,44,45], the hyaluronidase abnormal expression theory [46,47,48,49,50], the elastase abnormal expression theory [47,51,52], and the melanin over-synthesis theory [53,54,55,56,57] (Figure 1). Based on this premise, the present study critically examines the anti-photo-aging properties of marine bioactive peptides, encompassing their molecular characterization and underlying mechanisms associated with photoaging. The findings elucidate the protective attributes of marine bioactive peptides in mitigating photoaging and promoting skin well-being. The objective of this study is to explore the potential of marine bioderived products in promoting industrialization and the development of natural anti-photoaging agents for the food, pharmaceutical, and cosmetic industries. Additionally, this research aims to establish a theoretical foundation for the study and application of marine functionalized products. Ultimately, this investigation seeks to contribute to the understanding of marine bioactive peptides’ efficacy in combating skin photoaging, thereby providing valuable insights for future research in this field.

## 2. Anti-Photoaging Mechanism of Marine Bioactive Peptides

### 2.1. Peptide Anti-Skin-Photoaging by Inhibiting Oxidative Stress Damage

**Figure 2:**
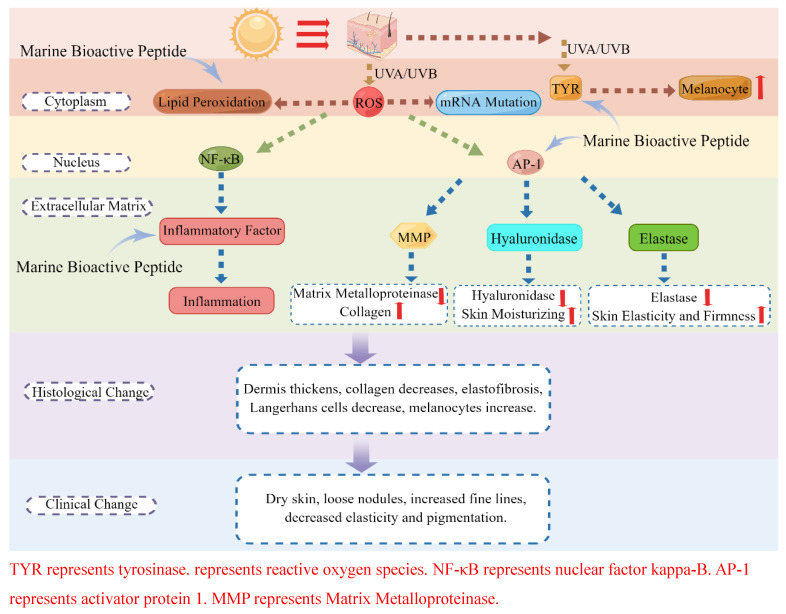
Schematic diagram of anti-photoaging mechanisms.

Oxidative stress, characterized by an imbalance between oxidation and antioxidant mechanisms within the body, constitutes a significant contributor to skin photoaging [58,59] (Figure 2). In the context of normal physiological conditions, a limited quantity of reactive oxygen species (ROS) exerts an immune defense effect and confers benefits to the body [60] (Table 1). However, excessive ROS production induced by ultraviolet radiation disrupts the cellular REDOX capacity, thereby impairing the oxidative stress defense system [61]. Consequently, the regulation of ROS levels assumes paramount importance in preserving the equilibrium of skin homeostasis [62].

Marine-derived peptides have garnered considerable interest among researchers due to their potent antioxidant characteristics [63]. Specifically, the peptides ICRD and LCGEC, derived from *tuna* eggs, exhibit robust in vitro DPPH free-radical scavenging activity and effectively protect HaCaT cells from ultraviolet B (UVB) radiation by upregulating the expression of SOD and GSH-Px [21]. Additionally, the *abalone* peptide ATPGEG demonstrates the capacity to mitigate UVB-induced ROS levels in HaCaT cells and inhibit cellular DNA damage resulting from UVB exposure [22]. According to reports, *jellyfish* collagen exhibits notable antioxidant activity and holds significant promise for the development of nutritional health products [23]. In the context of UV-induced skin photoaging in mice, the application of *jellyfish* collagen hydrolysate (JCH) has been found to augment the protective effect by elevating the levels of superoxide dismutase (SOD) and glutathione peroxidase (GSH-Px) [24]. Similarly, the gelatin hydrolysate AMW derived from *salmon* skin has been observed to diminish malondialdehyde (MDA) content and enhance antioxidant enzyme and glutathione (GSH) levels while mitigating the oxidative damage inflicted by ultraviolet radiation on the skin [25]. Three antioxidant peptides, namely TCP3 (PKK), TCP6 (YEGGD), and TCP9 (GPGLM), derived from the *bonipjack* heart artery balls of Skipjack tuna, have been found to enhance the activity of SOD, CAT, and GSH-P, effectively eliminating reactive oxygen species (ROS) and reducing intracellular malondialdehyde (MDA) levels [28]. Consequently, the protective capacity of HaCaT cells against UVB irradiation has been significantly enhanced [26]. Moreover, the impact of *tilapia* gelatin peptides on UV-induced skin damage in mice has also been investigated [27]. The findings demonstrate that the *tilapia* gelatin peptide LSGTGP can effectively neutralize hydroxyl radicals, thereby preventing UV-induced damage [27].

### 2.2. Peptide Anti-Skin-Photoaging via Anti-Inflammation

When cells are exposed to UV radiation and other environmental stimuli, they have the ability to release a group of small molecular peptides or glycoproteins known as cytokines, which play a crucial role in regulating the inflammatory response [64,65] (Figure 2). In normal physiological conditions, the production of cytokines remains at low levels, thereby avoiding any harm to the cells [66]. However, following exposure to ultraviolet irradiation, both epidermal and dermal cells can activate NF-κB, leading to the synthesis and secretion of inflammatory factors such as IL-1, IL-6, cyclooxygenase-2 (COX-2), and TNF-α, consequently inducing an inflammatory response [67]. Simultaneously, the presence of these cytokines induces mitochondrial impairment, leading to heightened levels of reactive oxygen species (ROS) and subsequent augmentation in the release of inflammatory mediators [68].

The potent anti-inflammatory properties of marine peptides have been extensively documented in the scientific literature [69]. Specifically, gelatin hydrolysate derived from the skin of *Pacific cod* has been found to effectively mitigate inflammation caused by UV radiation [38]. This is achieved through the suppression of pro-inflammatory cytokines IL-1α and TNF-α, thereby preventing UV-radiation-induced skin damage. The polypeptide (WNLNP) extracted from *oyster* protein can significantly down-regulate the inflammatory pathway of MAPK/NF-κB and reduce the overexpression of bax, which has a good protective effect against skin injury [70]. The hydrolysate (PWG) extracted from *Pacific cod* skin reduced the cytokines TNF-α, IL-6, and IL-1β associated with inflammation and inhibited inflammation by inhibiting the nuclear factor-κB (NF-κB) pathway, suggesting that PWG may be an effective anti-photoaging material [71]. Additionally, the impact of hydrolyzing six collagens extracted from *sturgeon* skin on photodamage induced by UVB radiation has been investigated [39]. The study revealed that the peptides DPFRHY and PEG, derived from *M. maritima*, effectively suppressed the abnormal expression of pro-inflammatory cytokines IL-1β, IL-6, TNF-α, and Cox-2. Specifically, DPFRHY demonstrated notable anti-inflammatory and cell repair properties [39]. Furthermore, PEG exhibited the inhibition of IL-1β, IL-6, PGE, TNF-α, and COX-2 production while also providing enhanced protection against UV-induced photoaging in mice [40]. The polypeptide (SEP) extracted from *by-products* of bonito fish can significantly reduce the expression levels of IL-6, IL-10, and TNF-α and has a good anti-inflammatory effect [72]. In addition, it has also been reported that SEP-3 significantly inhibits the inflammatory pathway of NF-κB [72], which has a strong potential to prevent photoaging and inflammatory diseases. Meanwhile, peptides isolated from marine actinomycetes have attracted attention for their unique biological activities [73]. The cyclic peptide isolated from Streptomyces maritimus CNB-091 can be used as an anti-inflammatory agent [74], while the peptide isolated from Streptomyces maritimus CNB-982 also has good anti-inflammatory activity and is expected to be used as an anti-photoaging agent [75]. However, it is important to note that limited research has been conducted in this particular field, thus necessitating further investigation.

### 2.3. Peptide Anti-Skin-Photoaging via Inhibition of Matrix Metalloproteinases

Under typical physiological circumstances, matrix metalloproteinases (MMPs) exhibit minimal expression within the human body [76] (Figure 2). However, their expression escalates swiftly upon exposure to various stimuli such as ultraviolet (UV) radiation, inflammation, and cancer [77]. Specifically, UVB radiation has been observed to induce the secretion of MMPs in a dosage-dependent manner [78]. This augmented expression of MMPs facilitates the degradation of the dermal extracellular matrix (ECM), particularly type I and type III procollagen, while concurrently impeding collagen synthesis [79,80,81]. As a result, these processes contribute to the desiccation and diminished elasticity of the skin [79,80,81]. Consequently, the inhibition of MMP expression and the promotion of collagen synthesis are crucial strategies in combating photoaging [82].

Inhibiting the aberrant expression of MMPs represents a significant approach in investigating the effects of skin anti-photoaging [83]. Notably, the peptides derived from the skin gelatin hydrolysate of *Pacific cod* (GEIGPSGGRGKPGKDGDAGPK and GFSGLDGAKGD) exhibited inhibitory properties against MMP-1 expression in mouse skin fibroblasts subjected to UV radiation [41]. By suppressing MMP-1 activity, the process of skin photoaging can be ameliorated. Additionally, peptides obtained from *Pinctada martensii* meat have been found to mitigate UVB-radiation-induced damage in HaCaT cells by inhibiting the expression of MMPs [42]. The levels of interstitial collagenase (MMP-1) and stromal lyase (MMP-3) were found to be reduced, leading to an improvement in UVB-induced cell damage in HaCaT cells. Peptides derived from *Pyropia yezoensis* (specifically peptide PYP1-5) were observed to inhibit the expression of the MMP-1 protein, thereby mitigating skin aging and demonstrating potential in combating photoaging [43]. Additionally, the enzymatic hydrolysate (OAH) obtained from *oyster* exhibited the ability to suppress the expression of MMP-1 and alleviate UV-induced cytotoxicity, thus exhibiting anti-photoaging properties [44]. The application of *tlapia* collagen hydrolysate YGDE resulted in a reduction in the enzymatic activity of MMP-1 and MMP-9, mitigated cellular damage induced by ultraviolet rays (UVB), and ameliorated the effects of skin photoaging [45].

### 2.4. Peptide Anti-Skin-Photoaging via Inhibition of Hyaluronidase

Hyaluronic acid, a biopolymer constituent of the dermal extracellular matrix, is naturally present in various tissues of the body, such as the synovial fluid, eyes, gums, bone tissue, and heart valves [84,85]. Its capacity to bind water enables it to contribute to the preservation of skin moisture and serves a significant function in skin rejuvenation by enhancing viscosity and reducing extracellular fluid permeability [86]. In the context of normal skin, hyaluronic acid synthesis is responsible for maintaining skin moisture [87]. Nevertheless, the reduction in hyaluronic acid levels is attributed to the excessive production of hyaluronidase [88] (Figure 2). Consequently, the inhibition of hyaluronic acid degradation is imperative for safeguarding the integrity of the skin.

The impact of hyaluronidase on skin photoaging has garnered significant scholarly interest [89]. Research has indicated that peptides derived from a variety of microalgae species (including *Sukka’s algae*, *Dunaliella*, and *Nanophyllum*) possess the ability to diminish hyaluronidase activity [46]. Specifically, peptides extracted from three microalgae species (*Dunaliella tertiolecta*, *Tetraselmis suecica*, and *Nannochloropsis* sp.) exhibit inhibitory effects on hyaluronidase [47]. Simultaneously, previous studies have provided evidence that the peptide levels derived from the maximum biomass of spirochete, namely pepsin (PHP), Subtilin A (PHA), and both PHS enzymes, possess anti-hyaluronidase activities [48]. Furthermore, the collagen-derived peptides from *squid (Todarodes pacificus)* generated through alkaline enzymes exhibit dose-dependent characteristics and display promising efficacy as anti-photoaging agents [49]. The administration of low-molecular-weight collagen peptides derived from fish scales has been demonstrated to stimulate the synthesis of hyaluronic acid in HaCaT cells. This process counteracts photoaging damage by upregulating the expression of the hyaluronate synthase 2 (HAS2) gene and downregulating the expression of the hyaluronase 1 (HYAL1) gene, thereby promoting improvement [50].

### 2.5. Peptide Anti-Skin-Photoaging via Inhibition of Elastase

Elastin, an extracellular matrix protein, imparts elasticity and resilience to various connective tissues including the aorta, lungs, cartilage, elastic ligaments, and skin [90]. In comparison to collagen, elastin exhibits a significantly higher degree of flexibility, approximately 1000 times greater [91]. Consequently, the principal role of elastin lies in conferring tissue elasticity [92]. The synthesis and secretion of elastin occur through the activity of vascular smooth muscle cells and fibroblasts [93]. This physiological phenomenon typically ceases shortly after the onset of puberty, coinciding with the maturation of the body. Alongside collagen, the production of elastin is initiated to uphold the suppleness and tautness of the skin [91]. Nevertheless, an excessive production of elastase leads to a decline in elastin fibers, thereby compromising the mechanical characteristics of the tissue (Figure 2). Consequently, the inhibition of elastase becomes imperative in safeguarding the integumentary system [94].

Marine bioactive peptides have demonstrated their potential in effectively inhibiting elastase activity within skin anti-photoaging pathways [42,89]. Previous studies have reported the beneficial impact of *squid* skin collagen hydrolysate in inhibiting elastin and serving as an effective anti-photoaging agent [51]. Notably, two peptides derived from *bonito*’s elastin hydrolysate, namely TGVLTVM and NHIINGW, have exhibited protective effects against UVA-irradiation-induced skin damage by effectively inhibiting elastase [52]. Furthermore, additional peptides with elastase inhibition properties have been identified from *Duneria*, *Susica*, and *Nannochloropsis* sp. [47]. This implies that the utilization of these peptides may potentially enhance skin health by preventing the deterioration of the protein matrix within the skin, although the precise mechanism has yet to be fully understood.

### 2.6. Peptide Anti-Skin-Photoaging via Inhibition of Melanin Over-Synthesis

Melanin serves as the primary protective mechanism against ultraviolet radiation in the skin [95]. Following exposure to UVB, melanocytes situated in the basal layer of the skin generate an excessive amount of melanin, leading to the manifestation of skin pigmentation [96] (Figure 2). The formation of this pigment is facilitated by a series of oxidation reactions mediated by the enzyme tyrosinase (TYR), with the synthesis, transportation, and catalytic activity of TYR playing pivotal roles in melanin synthesis [97]. Hence, directing attention towards the active constituents of marine bioactive peptides towards melanocytes, impeding the overproduction of melanin, and exploring novel tyrosinase inhibitors have emerged as a viable approach to mitigate the manifestations associated with skin photoaging.

The TYR inhibitory peptide derived from marine organisms exhibits the ability to hinder melanin production and enhance skin lightening, thereby demonstrating promising prospects in combating skin photoaging [98]. Notably, research has demonstrated that the protein hydrolysate obtained from the shrimp by-product *Kirin polysavone* exhibits significant TYR inhibitory activity [56]. This inhibitory effect is concentration-dependent, with a complete TYR inhibition observed at a concentration of 400 μg/mL [54]. Additionally, *tilapia* scale polypeptides possess the capacity to chelate copper ions, thereby affecting TYR activity [55]. In vitro investigations have demonstrated the potent inhibition of TYR activity and effective reduction of melanin synthesis in mouse melanoma cells through the utilization of polypeptide hydrolysates derived from *tilapia* by-products [55]. Furthermore, there have been numerous in vivo and in vitro evaluations of the use of active collagen peptides derived from fish by-products. Notably, the marine bioactive peptide DLGFLARGF has exhibited the ability to impede tyrosinase activity, consequently hindering melanin production [57]. The squamosal fish’s collagen peptide demonstrates a remarkable moisture absorption capacity of 20%, effectively inhibits tyrosinase activity and melanin synthesis, and exhibits promising anti-photoaging properties [53].

## 3. Skin Protective Effects of Marine Bioactive Peptides

### 3.1. Peptides Improve Skin via Photoprotective Mechanisms

Skin aging encompasses both intrinsic aging and photoaging, with research indicating that photoaging contributes to over 80% of facial aging [99]. External factors that contribute to skin photoaging primarily consist of ultraviolet (UV) radiation, infrared radiation, chemical smoke, dust, and haze, with UV radiation being the most influential [100]. Numerous studies have demonstrated the efficacy of marine biopeptides in combating photoaging, making them a promising ingredient for the development of cosmeceuticals aimed at reducing skin aging.

The present study investigated the anti-photoaging effects of the peptide LSGYGP, isolated from the skin of *tilapia* [27]. It was observed that this peptide exerted its beneficial effects on the skin by utilizing its antioxidant activity to ameliorate UV-induced photoaging in mice [27]. In addition, it was found that *cod* skin gelatin hydrolysate (CGH) can inhibit MMP-1 and contribute to its anti-photoaging expression [41]. Protein hydrolysates derived from various sources such as fish bones, scales, and digestive organs were also identified as potential agents for improving skin aging [101,102,103]. Research findings indicate that *starfish* collagen peptides possess the ability to diminish the expression of MMP-1, which is induced by UV radiation photoaging, thereby exhibiting anti-photoaging properties [44]. Polypeptides isolated from *scallops* can inhibit UVA-induced ROS production and protect HaCaT cells from UVA-induced apoptosis [70]. Polypeptide (JCH) extracted from *jellyfish* (*Rhopilema esculentum*) mitigated abnormal UV-induced changes in antioxidant defense systems such as superoxide dismutase and glutathione peroxidase, effectively protecting skin from UV radiation [104]. The hydrolysate (PWG) extracted from *Pacific cod* skin decreased the cytokines TNF-α, IL-6, and IL-1β associated with inflammation and increased the contents of antioxidant enzymes, HO-1, SOD, GPx, CAT, and GSH. These multi-target mechanisms suggest that PWG may be an effective anti-photoaging material [71]. Additionally, the UVB irradiation of human immortalized keratinocytes (Hacats) and mouse aging models have been studied. The hexapeptide (AAH) extracted from *spirulina* can significantly increase the expression of SOD and GSH-P and reduce the expression of MMP-1 and MMP-3, which has potential applications in preventing skin photoaging [105]. The derived peptide (CDP) extracted from *Chlorella* can inhibit UVB-induced MMP-1 expression in skin fibroblasts and achieve photoprotection [106].

### 3.2. Peptides Improve Skin via Anti-Microbial Mechanisms

The skin is frequently exposed to environmental factors and can be harmed by microbial agents and ultraviolet radiation [107]. The aging process of the skin diminishes the production of protective bacteria, resulting in skin damage [108]. Consequently, it is imperative to investigate bioactive peptides that exhibit resistance against various bacteria such as *Staphylococcus aureus*, *propionibacterium acnes*, *Pseudomonas aeruginosa*, *Enterococcus faecium*, *Acinetobacter baumannii*, *Klebsiella pneumoniae*, *propionibacterium acnes*, and *Escherichia coli*. Numerous studies have demonstrated the efficacious antibacterial activity of marine-derived biopeptides, thereby highlighting their considerable potential for application in the realm of skin protection.

Marine antimicrobial peptides exhibit a diverse array of antibacterial and bactericidal properties, rendering them suitable for employment as fungicides [109]. Moreover, these peptides possess substantial potential in the realm of skin protection [110]. They are extensively present in various marine fish species [111], such as *Capitella teleta*, *Porphyra yezoensis*, *Octopus minor*, *Olivancillaria hiatula*, *Mytilus coruscus*, *Green tiger shrimp* (*Peaneaus semisulcatus*), *Hypoptychus dybowskii*, and *Cyanobacteria*, demonstrating notable efficacy against bacterial pathogens (Table 2) [112,113,114,115,116,117,118]. The proteolytic peptides derived from *S. longicruris*, a brown seaweed, exhibited notable antibacterial efficacy and demonstrated significant activity against *Gram-positive Staphylococcus aureus* [119]. Specifically, HAHp2-3-I, consisting of five cationic peptides (MLTTPPHAKYVLQW, SHAATKAPPKNGNY, PTAGVANALQHA, QLGTHSAQPVPF, and VNVDERWRKL) and obtained from the hydrolysate of *semi-engraan* pepsin, displayed robust resistance against *Escherichia coli* [8]. Additionally, a separate investigation revealed that the half anchoa pepsin hydrolysate (HAHp) exhibited a potent inhibitory effect against *Escherichia coli*, suggesting its potential as a protective agent for the skin [120]. The potential of protamex hydrolysate, derived from *Atlantic mackerel*, to provide skin protection through the inhibition of both *Gram-positive* (*intrinsic listeria*) and *Gram-negative bacteria* (*Escherichia coli*) has been observed in various studies [121]. Furthermore, these studies have consistently shown that the inhibitory activity against both *Gram-positive* (*intrinsic listeria*) and *Gram-negative bacteria* (*Escherichia coli*) is more pronounced in Atlantic mackerel [122].

### 3.3. Peptides Improve Skin via Skin Repair

Skin is an important immune organ of the human body, but it easily experiences health problems under the influence of physiological factors and external environmental factors. At the same time, as the efficacy of collagen peptides in improving the skin has become apparent, the research in this area has been increasing in recent years (Table 3). Studies have shown that the ingestion of collagen peptides inhibits UVB-induced reduced skin hydration, epidermal hyperplasia, and decreased soluble type I collagen. These results suggest that collagen peptides as a dietary supplement may be beneficial in inhibiting UVB-induced skin damage and photoaging [123]. The oral administration of marine collagen peptides from salmon skin promoted skin wound healing and angiogenesis in rats in [124]. Meanwhile, the oral administration of marine collagen peptides from *salmon* skin also promoted skin wound healing in rats in [125]. *Cod* skin collagen polypeptides have good moisture absorption and moisture retention properties and can reduce the damage from ultraviolet light on the skin [126]. At the same time, some studies have shown that *cod* skin gelatin peptides can inhibit the production of melanin [127]. Paralichthys olivaceus (PO) and Alaska pollock Gadus chalcogrammus (AP) proteolytic substance increased the viability of UVB-irradiated HaCaT cells and decreased the intracellular and extracellular melanin content of stimulated B16F10 cells. These results indicate that PO and AP have potential applications in the cosmetics industry [128]. In a UVB-irradiated HDF cell model, Pacific cod protein hydrolysate (PWG) had a protective effect against photoaging by down-regulating MMP1 [71]. In addition, there are clinical trials showing that marine collagen peptide (MCP) can improve skin properties without the risk of oxidative damage [125].

The reparative properties of marine biological collagen and its hydrolysate for skin damage caused by UV exposure have been observed [129]. In vitro cell experiments have demonstrated that the hydrolysate derived from *sponge* collagen possesses wound healing capabilities and tissue repair functions for UV-irradiated fibroblasts and keratinocytes [130]. Additionally, scholars have conducted mouse experiments and discovered that gelatin hydrolysate from *trilefish* exhibits a reparative effect on UV-induced skin damage [25]. The reparative properties of *jellyfish* collagen peptide have been observed in its ability to restore endogenous collagen and elastin fibers in compromised skin [104]. A study conducted on mice with cortical injury demonstrated that the administration of *nude sidereal* collagen peptide resulted in successful wound healing [131]. Furthermore, the application of marine collagen peptides (MCPs) derived from *salmon* skin exhibited a substantial enhancement in the tensile strength of skin wounds in rats [132]. The specific peptides employed in cosmetic formulations exhibit diverse effects. Notably, collagen peptides derived from deep-sea fish possess a range of functionalities [133], such as skin whitening, freckle removal, moisturization, nutritional repair, and anti-aging properties. Consequently, these peptides hold significant promise in safeguarding the skin.

## 4. Bioavailability of Marine Bioactive Peptides

**Figure 3:**
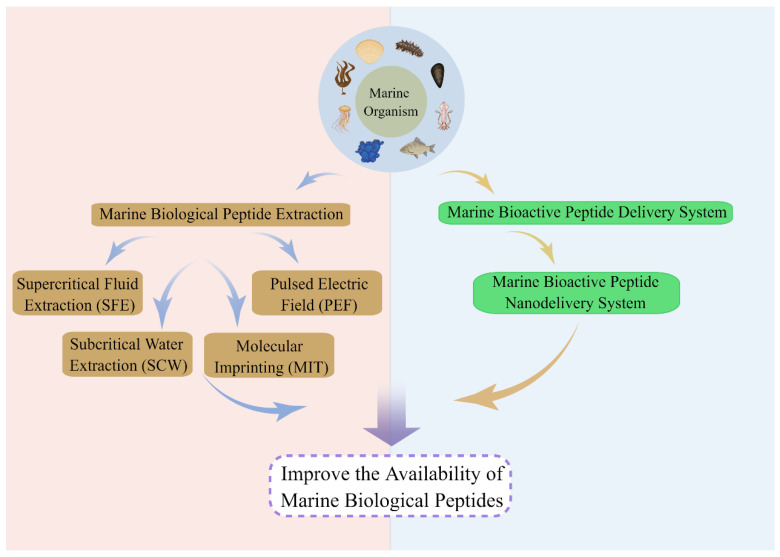
Marine bioactive peptide bioavailability enhancement pathway.

The photoaging of the skin is a notable characteristic associated with the aging process, particularly concerning women [134]. Statistical data reveal that a mere 2% of Chinese women undertake anti-aging measures, while the anti-aging market in China reached a substantial value of RMB 6.4 billion in 1990 [135]. In addition, by 2027, the global market for skin whitening and anti-aging products is expected to reach USD 1.23 billion [136] and USD 83.2 billion [137], respectfully. Marine bioactive peptide substances exhibit potent anti-photoaging and skin protection properties with minimal toxicity; however, the investigation into their underlying mechanisms remains at a nascent stage. Therefore, it is imperative to undertake the isolation and purification of marine bioactive peptides, as well as to conduct extensive investigations into their safety and bioavailability. Additionally, there is a need to further explore the relevant indicators and pathways associated with photoaging. It is crucial to note that the development of marine bioactive peptide drugs is still in its nascent stage and lacks clinical application. Consequently, enhancing the bioavailability of marine bioactive peptides through increased modifications and more favorable methods of separation and purification has become highly significant. Therefore, efforts should be made to improve the separation and purification techniques for marine bioactive peptides in order to enhance their bioavailability (Figure 3).

### 4.1. Improvement of Bioavailability of Marine Bioactive Peptides via Isolation and Purification

The majority of polypeptide bioactive compounds derived from marine organisms exist as intricate mixtures, and the presence of these complex constituents can impede the extraction procedure of polypeptide compounds. Consequently, prior to conducting an in-depth investigation of polypeptide compounds, it is imperative to effectively extract and purify these compounds from marine organisms to facilitate a more comprehensive and meticulous analysis. A fundamental requirement for identifying polypeptide compounds in marine organisms is the establishment of effective extraction technology. Consequently, the exploration of novel approaches for extracting polypeptide compounds with high purity has emerged as a new area of research. The conventional method of extracting and purifying polypeptides relies on the utilization of organic solvents, which is frequently associated with inefficiency, time consumption, and labor intensiveness [138]. Furthermore, the utilization of organic solvents is restricted in the food and pharmaceutical industries due to certain limitations. Moreover, the viability of their application is further compromised by the potential generation of degradation products during the extraction process. As scientific and technological advancements continue to unfold, numerous intricate extraction and purification methodologies have been developed and implemented, such as supercritical fluid extraction (SFE) [139], subcritical water extraction (SCW) [140], pulsed electric fields (PEFs) [140], and molecular imprinting technology (MIT) [141]. These technologies provide enhanced functionalities by mitigating the constraints of conventional extraction methods.

Supercritical fluid extraction (SFE) is a technique wherein a supercritical fluid is employed as a solvent to facilitate the separation of a mixture, owing to the notable permeability and solubility of the fluid in this state. In contrast to conventional toxic, flammable, and volatile organic solvents, SFE predominantly employs carbon dioxide as an extractant [142]. SFE technology holds significance in the extraction of marine polypeptides. Moreover, it possesses the ability to effectively isolate and extract various other marine biological active substances, including marine biotoxins, essential oils, marine natural pigments, and select rare amino acids [143].

Subcritical water extraction (SCW) refers to the treatment of liquid water under high pressure at temperatures above its boiling point (100–374 °C). This process utilizes subcritical water as a solvent for both polar and non-polar compounds due to its lower dielectric constant in the subcritical state, which enhances its affinity for less polar compounds and facilitates excellent protein solubility [144]. Furthermore, the application of a high temperature and pressure during SCW also triggers protein hydrolysis, leading to the generation of peptides and amino acids. The SCW process resulted in a peptide yield of 87.4% from *sardine* by-products [144]. Additionally, the hydrolysis rate of *squid* viscera treated with SCW reached 95% [145]. By employing SCW in the extraction process, the need for enzyme and acid–base ion removal is eliminated, thereby establishing SCW as an environmentally friendly technology for enhancing the yield of marine bioactive peptides [146].

The utilization of pulsed electric field (PEF) technology has gained prominence in the fields of the low-temperature sterilization and preservation of agricultural products. In recent times, there has been a growing trend towards employing PEF for the extraction of bioactive substances from food-source substrates. PEF facilitates the enhancement of membrane permeability through mechanisms such as membrane electroporation or electroosmosis, thereby enabling the release of intracellular proteins and exogenous enzymes. Consequently, the integration of PEF with enzymatic or solvent extraction methods presents a promising approach to augment the production yield of marine bioactive peptides [147]. For instance, the utilization of a pulsed electric field (PEF) in conjunction with enzymes, such as flavor enzymes and trypsin, during the treatment of *abalone* (*Haliotis discus hannai Ino*) viscera resulted in a significantly higher yield of hydrolysate compared to extraction using a single enzyme [148]. Furthermore, the application of PEF to *senedesmus* almeriensis demonstrated enhanced enzymatic (specifically alkaline enzyme) hydrolysis, leading to an increase in the yield of bioactive peptides from 40.8% to 50.6% [149]. The utilization of a pulsed electric field (PEF) in marine bioprocessing remains constrained primarily by equipment costs. Nevertheless, the inherent attributes of PEFs, such as their low energy consumption, rapid processing time, and comparatively gentle extraction conditions, hold significant promise for augmenting the production capacity of bioproducts derived from marine organisms.

Molecular imprinting (MIT) is a novel interdisciplinary technique that integrates receptor–antibody mechanisms and expertise in biochemistry, structural chemistry, and materials chemistry [150]. Due to its remarkable selectivity, robust stability, and broad applicability, MIT has garnered significant attention and extensive investigation in recent years [149]. The advancement of molecular imprinting technology holds the potential to facilitate the efficient and expeditious separation of biomolecules, including polypeptides and proteins, from intricate mixtures [149]. However, during the preparation procedure of molecularly imprinted polymers, it is typically imperative to incorporate biomolecules as imprint templates, some of which possess conformational flexibility, volatility, and inactivation.

### 4.2. Improvement of Bioavailability of Marine Bioactive Peptides via Nanodelivery Systems

The potential disparity between the activity of peptides in vitro and in vivo is influenced by the presence of enzymes and stomach acids during gastrointestinal digestion, which can impact peptide bioavailability. Consequently, it is imperative to carry out both in vitro and in vivo experiments to ascertain the validity of these findings. In particular, the outcomes of in vivo studies need to be verified in order to confirm the observed results. In the event that biological activity is compromised in vitro, it is essential to re-evaluate peptide concentrations or matrix properties prior to conducting in vivo testing. In order to ascertain the gastrointestinal digestibility and solubility, absorption, distribution, and utilization of each peptide, as well as to determine the required dosage for achieving its efficacy, it is imperative to conduct in vivo studies encompassing both animal and human subjects [151]. Various models, including invertebrate *C. elegans* and *fruit flies*, vertebrate rats and mice, and human subjects, have been employed for in vivo investigations of peptide functionality, particularly in the context of functional foods. In vivo experiments serve as a reliable means of observing the potential outcomes associated with the consumption of a substrate containing peptides. However, these experiments are intricate and costly, necessitating the involvement of either animals or humans and often requiring an extended duration. To date, there has been a scarcity of research conducted on the bioavailability of marine compounds.

Despite the demonstrated diverse biological activities of marine bioactive peptides, their practical application is hindered by challenges such as hydrophobicity, chemical instability, and limited bioavailability. Consequently, the preservation of peptide activity post-digestion necessitates the exploration of protective measures. Among these measures, nanodelivery has emerged as the most extensively investigated and documented method.

Nanotechnology has emerged as a promising strategy for addressing the challenges associated with incorporating bioactive peptides into food [151]. Recent studies have demonstrated the efficacy of utilizing nanosized carriers, such as nanoemulsions, nanoliposomes, microemulsions, micelles, nanostructured lipid carriers, solid lipid nanoparticles, and polymer nanoparticles, to encapsulate hydrophobic bioactive peptides. These encapsulated peptides have exhibited enhanced biological effects, highlighting the potential of nanotechnology in developing effective delivery systems for bioactive peptides in food [152]. Furthermore, nanometer-based delivery systems possess the ability to modify the uptake pathway of bioactive substances by manipulating their metabolism and bioactivity within an organism [153]. This enhanced biological performance can be attributed to the diminutive size, augmented surface area, and refined surface chemistry of these systems. For instance, the encapsulation of peptide grades derived from fish skin gelatin within a nanoliposome system resulted in a prolonged release rate, decreasing from 41% to 24% over a span of 30 h, accompanied by a comparatively elevated antioxidant activity ranging from 15.7% to 74.7% when compared to unencapsulated peptide grades [154]. In two additional autonomous investigations, the nanoencapsulation of fish protein peptides was achieved through ion-to-gel techniques, yielding improved gastrointestinal stability and bioavailability outcomes [155]. Nevertheless, the safety considerations associated with nanoencapsulated marine peptides should not be disregarded. Ultimately, comprehending the destiny and conduct of nanoparticles in food and the human organism is imperative for safety evaluation, with their capacity to maintain structural integrity within the gastrointestinal tract serving as the principal determining factor. In the presence of digestive enzymes, strong acids, and bile salts, nanoparticles have a tendency to aggregate or experience alterations in size, consequently impacting their capacity to be absorbed and traverse biological barriers within the body [156]. Moreover, nanoparticles derived from digestible organic substances, such as proteins, lipids, or starches, present a lesser risk compared to nanoparticles obtained from indigestible inorganic materials, such as metal or metal oxide nanoparticles.

## 5. Conclusions and Prospects

The investigation of marine active peptides constitutes a crucial domain within marine research and development in the 21st century, currently experiencing rapid advancements and yielding significant outcomes. Nevertheless, the present research endeavors in this area are insufficient within our nation, necessitating an augmentation in research funding for marine peptides. This would enable the further examination and elucidation of the mechanism of action of marine peptides, the exploration of the inherent characteristics of active peptides, and the fortification of the application research pertaining to marine active peptides across diverse domains.

Marine bioactive peptides have emerged as a significant asset in combating skin photoaging. Extensively investigated for their diverse biological attributes, including theories related to oxidative stress, the abnormal expression of matrix metalloproteinase, inflammatory response, the abnormal expression of hyaluronidase, the abnormal expression of elastase, and the excessive synthesis of melanin, these peptides have garnered considerable attention for their potential in enhancing skin health. Novel extraction methods, such as supercritical fluid extraction (SFE), subcritical water extraction (SCW), pulsed electric fields (PEFs), and molecular imprinting technology (MIT), have successfully yielded several bioactive peptides from marine fish. These methods are regarded as safer alternatives for the development of cosmeceutical products. Simultaneously, the utilization of marine bioactive peptides aids in mitigating environmental pollution resulting from waste generated by the fish processing industry. Furthermore, the potential topical applications or oral utilization of marine bioactive peptides for safeguarding the skin highlight their significant biological activities (Table 4). However, it is crucial to acknowledge that despite the immense potential of marine fish-derived proteins and peptides in the field of cosmeceuticals, the majority of these compounds remain in the experimental phase. Consequently, additional investigations pertaining to their formulations and long-term safety are imperative for their successful commercialization. Additionally, it is imperative to explore the development of supplementary products that can enhance the bioavailability and efficacy of proteins and peptides derived from marine sources, thereby augmenting their potential in the field of cosmeceuticals, particularly in terms of tissue regeneration.

In summary, researchers can obtain a large number of bioactive peptides with strong light protection and light repair properties from marine organisms, which have a series of important functions such as blocking light penetration, anti-oxidant activity, anti-inflammatory behavior, damage repair, delaying degradation, promoting synthesis, and stabilizing the skin barrier and can be applied to various scenarios such as external use, oral use, and product addition. This is of great value for future research in light damage and primary skin disease prevention, product development, and other fields. In addition, marine bioactive peptides, which are generally more acceptable due to their “natural and healthy” characteristics, can promote a positive response from consumers, provide social impetus, and help to further tap the great potential of ocean treasures, providing valuable insights for future research in this field and thus laying a solid foundation for the global anti-aging molecule market.

## Figures and Tables

**Table 1:** Potential bioactive antioxidant peptides from marine resources.

Source	Enzyme Used	Peptides (Amino Acid Sequence)	Mechanism of Action	In Vivo or In Vitro	Reference
*Tuna* eggs	-	Ile-Cys-Arg-Asp and Leu-Cys-Gly-Glu-Cys	Inhibition of DPPH radicals and activation of SOD and GSH-Px	in vivo	[21]
*Boiled abalone* by-products	-	Ala-Thr-Pro-Gly-Asp-Glu-Gly	Inhibition of ROS radicals	in vitro	[22]
*Jellyfish* collagen	Pepsin	-	Activation of total antioxidant activity	in vitro	[23]
*Rhopilema esculentum*	Pepsin	-	Activation of SOD, CAT, and GSH-Px	in vivo	[24]
*Salmon* skin	-	-	Activation of SOD, CAT, and GSH-Px	in vivo	[25]
*Katsuwonus pelamis*	-	TCP3, TCP6, and TCP9	Activation of SOD, CAT, and GSH-Px	in vitro	[26]
*Tilapia* gelatin	-	Leu-Ser-Gly-Tyr-Gly-Pro	Scavenging free radicals	in vitro	[27]
*Katsuwonus pelamis*	-	-	Scavenging free radicals	in vitro	[28]
*Monkfish*	Trypsin	Glu-Trp-Pro-Ala-Gln, Phe-Leu-His-Arg-Pro, and Leu-Met-Gly-Gln-Trp	Inhibition of DPPH radicals and hydroxyl radicals; activation of SOD, CAT, and GSH-Px	in vitro	[29]
*Macroalga P. palmata*	Corolase PP	Ser-Asp-Ile-Thr-Arg-Pro-Gly-Gly-Asn-Met	Activation of oxygen radical absorption capacity (ORAC) and iron reduction antioxidant capacity (FRAP)	in vitro	[30]
*Thunnus obesus*	Alcalase, α-chymotrypsin, neutrase, papain, pepsin, and trypsin	H-Leu-Asn-Leu-Pro-Thr-Ala-Val-Tyr-Met-Val-Thr-OH	Inhibition of DPPH, hydroxyl, superoxide, and alkyl radicals	in vitro	[31]
*Magalaspis cordyla*	Pepsin/trypsin, and α-chymotrypsin	Asn-His-Arg-Tyr-Asp-Arg	Inhibition of DPPH and hydroxyl radicals	in vitro	[32]
*Otolithes ruber*	pepsin/trypsin and α-chymotrypsin	Gly-Asn-Arg-Gly-Phe-Ala-Cys-Arg-His-Ala	Inhibition of DPPH and hydroxyl radicals	in vitro	[32]
*Hypoptychus dybowskii*	Alcalase, neutrase, α-chymotrypsin, papain, pepsin, and trypsin	Ile–Val–Gly–Gly–Phe–Pro–His–Tyr–Leu	Inhibition of DPPH radicals	in vitro	[33]
*Oreochromis niloticus*	Alcalase, pronase E, pepsin, and trypsin	Asp-Pro-Ala-Leu-Ala-Thr-Glu-Pro-Asp-Pro-Met-Pro-Phe	Inhibition of DPPH, hydroxyl, and superoxide radicals	in vitro	[34]
*Decapterus maruadsi*	Alcalase, neutral protease, papain, pepsin, and trypsin	His-Asp-His-Pro-Val-Cys and His-Glu-Lys-Val-Cys	Inhibition of DPPH and hydroxyl radicals	in vitro	[35]
*Johnius belengerii*	Trypsin, R-chymotrypsin, and pepsin	His-Gly-Pro-Leu-Gly-Pro-Leu	Inhibition of DPPH radicals	in vitro	[36]
*Paralichthys olivaceus*	Papain, pepsin, trypsin, neutrase, alcalase, kojizyme, protamex, and α-chymotrypsin	Val-Cys-Ser-Val and Cys-Ala-Ala-Pro	Inhibition of DPPH radicals	in vitro	[37]

**Table 2:** Potential bioactive antimicrobial peptides from marine resources.

Source	Enzyme Used	Peptides (Amino Acid Sequence)	Microorganisms	Reference
*Capitella teleta*	-	-	*E. coli* BL21	[110]
*Porphyra yezoensis*	Pepsin	Thr-Pro-Asp-Ser-Glu-Ala-Leu	*Staphylococcus aureus*	[112]
*Octopus minor*	-	Gly-Trp-Leu-Ile-Arg-Gly-Ala-Ile-His-Ala-Gly-Lys-Ala-Ile-His-Gly-Leu-Ile-His-Arg-Arg-Arg-His	* Candida albicans *	[113]
*Olivancillaria hiatula*	-	-	* Pseudomonas aeruginosa *	[114]
*Mytilus coruscus*	-	-	* Gram-positive bacteria * —*Bacillus*, *Bacillus subtilis*, *Clostridium perfringens*, *Staphylococcus aureus*, *Streptococcus*, *Streptococcus mutans; Gram-negative bacteria—* *Escherichia coli*, *Pseudomonas aeruginosa*, *Vibrio alginolyticus*	[115]
*Green tiger shrimp* (*Peaneaus semisulcatus*)	-	-	* Staphylococcus aureus *	[116]
* Hypoptychus dybowskii *	-	Ser-Arg-Ser-Ser-Arg-Ala-Gly-Leu-Gln-Phe-Pro-Val-Gly-Arg-Ile-His-Arg-Leu-Leu-Arg-Lys	* Staphylococcus aureus and * *Escherichia coli*	[117]
*Cyanobacteria*	-	-	* Candida albicans *	[118]

**Table 3:** Potential skin-protective bioactive peptides from marine resources.

Source	Functional Product	Processing Method	Cosmeceutical Function	Reference
*Salmon* skin	Collagen peptides	Water, protease	Wound healing	[124]
Fish scales	Collagen peptides	Hot water, enzymatic	Improving skin elasticity	[125]
*Codfish* skin	Collagen polypeptides	Water, pepsin, and alkaline protease	Moisturizer, antioxidant	[126]
*Pacific whiting* skin	Hydrolysate gelatin	Hot water	Anti-photoaging, delayed skin wrinkling	[71]
*Pacific cod* skin	Gelatin and polypeptides	Hot water extraction, pepsin, and alkaline protease hydrolysis	Melanogenesis inhibition	[127]
*Olive flounder* and *Alaska pollock* skins	Fish skin hydrolysates	Enzymatic hydrolysis (pepsin, alcalase, protemax)	Minimizing ROS levels, enhancing the viability of UVB-irradiated HaCat cells and human dermal fibroblasts	[128]
Scales of *Tilapia zillii*	Polypeptides	Pepsin	Increasing skin hydration and decreasing epidermal hyperplasia	[123]

**Table 4:** Marine bioactive peptides as cosmetic ingredients.

Company	Country	By-Product Resource	Bioactive Compounds	Cosmeceutical Function	Reference
Rousselot	France	Fisk skin and bone	Collagen peptides	Skin moisturization, enhanced skin collagen density	[157]
Celergen Inc	Switzerland	Fish skin	Collagen hydrolysate	Enhanced skill elasticity	[125]
Abyss	France	Fish skin	Collagen hydrolysate	Reduced appearance of wrinkles	[158]
Finn Canada	Canada	Salmon skin	Collagen	Improved skin condition; treatment of various skin problems, such as wrinkles, spots, dryness, dullness, and acne	[159]
Kenney and Ross Limited	Canada	Fish skin	Collagen	Stimulates healthy skin, nails, and hair	[160]
Nuwen	France	Fish skin	Collagen hydrolysate	Skin moisturization	[161]
One Ocean	United States	Fish skin	Collagen	Skin moisturization, anti-wrinkle	[162]
Osteralia	France	Mother-of-pearl	Oyster shell	Anti-aging, skin nourishment	[163]

## Data Availability

Not applicable.
